# Modified total skin electron treatment for a paraplegic patient

**DOI:** 10.1002/acm2.70162

**Published:** 2025-07-14

**Authors:** Thomas Martin, Geoff Nelson, David Gaffney, Christian Dial, Martin Szegedi, Prema Rassiah

**Affiliations:** ^1^ Department of Radiation Oncology University of Utah Huntsman Cancer Hospital Salt Lake City Utah USA

**Keywords:** Decubitus, Mycosis Fungoides,Paraplegic, Radiation Therapy, Total Skin

## Abstract

Total Skin Electron Therapy (TSET) has been a highly effective treatment for mycosis fungoides (MF). However, the standard TSET treatment requires the patient to stand upright in six different positions for an extended time period, which may not be possible for some patients. Herein, is reported a modified TSET is reported to accommodate a paraplegic patient. Two dual beams of 6 MeV, commissioned for the standard TSET, were used for treatment. The patient's treatment surface was maintained within 190–240 cm isocenter to surface distance (ISD) and 100 cm horizontally. In vivo measurement showed that a ± 30% dose uniformity was achieved at the patient's surface.

## 1 INTRODUCTION

Total Skin Electron Therapy (TSET) is an established and effective modality in radiation therapy, designed to treat the entire surface of the skin. This therapy is primarily used for conditions like cutaneous lymphomas, particularly mycosis fungoides (MF)^[^ ^1^ ^]^ TSET uses low‐energy electron beams that penetrate only the top layers of the skin, typically up to 0.5–1.5 cm deep.

To ensure even coverage of the entire skin surface, patients are positioned using a standard set of anatomical poses, via the Stanford Technique, or a rotating platform.^[^ ^2^ ^]^ When utilizing these techniques, a dose uniformity of ± 10% is achievable at the patient surface.^[^ ^2^ ^]^

Certain areas, such as the eyes, hands, and feet, may be shielded during treatment; whereas areas that do not receive adequate radiation during TSET, such as the soles of the feet, perineum, and scalp, may be boosted with supplementary electron fields. Additionally, shielding may be utilized for areas that do not have disease involvement, such as the head, scalp, upper back, and so forth.^[^ ^3^ ^]^

Ultimately, clinical objectives of homogeneous dose delivery to the skin surface and shielding of sensitive or uninvolved areas necessitate several radiation fields and patient positions. Furthermore, primary fields are delivered at extended distances to increase field size, which reduces achievable dose rates and extends treatment times. Positions may be prohibitive for certain patient populations or difficult to tolerate for the duration of delivery. In this report, a case is presented where a patient‐specific modified TSET technique and patient positioning were applied to accommodate a paraplegic patient.

## 2 CASE PRESENTATION

A 41‐year‐old male presented with the chief complaint of a worsening skin rash present for 8 years. He reported treatment with topical steroids and over‐the‐counter creams with little improvement. Exam findings noted large oval eczematous plaques on his back, abdomen, and thighs. His face, ears, arms, and lower legs (below the knees) were not involved. Multiple biopsies were taken, and the diagnosis revealed cutaneous T‐cell lymphoma, mycosis fungoides at the patch/plaque stage. The patient was paraplegic from a spinal cord injury 22 years prior. After extensive workup, he was found to have MF covering 23.5% of his skin surface (18.7% patch, 4.8% plaque). Initial treatment resulted in a large plaque on his right posterior shoulder, which was treated with 6 MeV electrons (1 cm bolus) to 9 Gy in three fractions with a complete response. He subsequently was treated with oral acitretin and had a partial response (MF involving 12.2% of skin surface). He was referred back to radiation oncology, and TSET was recommended. He had significant patches on his chest, back, and upper legs. Based on the location of his disease, his paraplegia, and his permanent flexion of his lower extremities, it was decided to employ TSET in 12 Gy in eight fractions. No boosts were needed to other areas of the patient's skin that the modified technique would not be able to reach at this time.

The Stanford technique is used as a standard in our department, where six dual electron fields (two gantry positions each) and six patient positions spaced at 60‐degree interval are employed. For treatment with 6 MeV electrons, a 0.6 cm acrylic scattering insert at the linac head and a source skin distance of approximately 200 cm were used for treatment.

**FIGURE 1:**
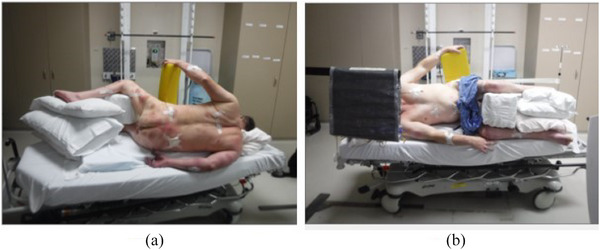
Shows the patient positioning for treatment (a) posterior decubitus and b. anterior decubitus with the patients face shielded.

Due to the patient's severe disabilities, the patient was unable to stand or lie prone, so the standard TSE approach was modified. Additionally, the patient did not present with disease on the left or right lateral portions of their skin. Therefore, the standard six dual fields were reduced to two dual fields only in the anterior and posterior positions, with the understanding that the lateral portions of the patient's skin would not receive the full dose. For this, the patient was positioned decubitus as shown in Figure 1. The face did not present with disease and was shielded with a 3 mm‐thick lead, as shown in Figure 1b. No additional shielding was prescribed to the feet, hands, fingertips, and so forth to reduce the dose to those areas. It was also explained to and accepted by the attending radiation oncologist that, due to the reduction from six dual fields to two dual fields, a compromise of the uniform dose would need to be accepted in order to treat this patient.

The patient positioning was divided into four main positions to average out the ISD positions of the arms, and thus average the dose to the arms. The positions of the patient were AP and PA decubitus with the patient lying on their right side, and AP and PA decubitus with the patient lying on their left side. Figure 1 shows the patient setup in the AP and PA directions while lying on their right side. The other position not shown is the AP and PA setup while the patient is laying on their left side. There were four fractions delivered while the patient lying on their left side, and the other four with the patient lying on their right side.

### 2.1 Pre‐treatment measurements

Previously, TSE treatments were commissioned using the six dual‐field Stanford technique to achieve ± 10% vertical uniformity on a Varian Truebeam linear accelerator. The depth of d_max_ for the dual field of high‐dose TSE 6 MeV was characterized to be 0.893 cm (measured in solid water). The R_50_ for the TSE 6MeV field is 1.72 cm. The photon contamination for the dual fields was measured to be approximately 3%.

**FIGURE 2:**
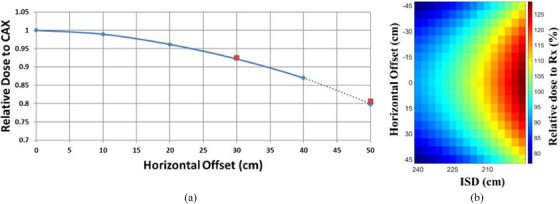
On the left, (a) shows the measured (blue solid line) and extrapolated relative dose to the central axis uniformity (blue dotted line). The red data points are the measured data taken from the 30 cm vertically below the central axis to verify the extrapolation. On the right, (b) shows the isodose plot relative to the prescription as a function of lateral offset and ISD. ISD, isocenter to surface distance.

Measurements were made to determine the horizontal uniformity (superior to inferior in the patient direction) since the patient was lying down instead of standing. Measurements were made using solid water and a Roos 34001 (PTW Freiburg, Germany) parallel plate ion chamber. The horizontal uniformity was measured by taking measurements of the dual fields (65 degrees and 115 degrees gantry angle) at d_max_ in the solid water phantom at a treatment distance of 230 cm ISD at the central axis. Measurements were then made at 10 cm increments horizontally up to 40 cm off the central axis, at 30 cm below the central axis in the vertical direction. The selected measurement height of 30 cm below the central axis, with the gantry positioned at 90 degrees, corresponds to the patient's level. This was repeated for ISD 190 cm. The data was extrapolated up to 50 cm using a least‐squares polynomial fit. From the measurements, it was shown that horizontal uniformity was within ± 20% of the CAX dose (Figure 2a).

A verification measurement at 50 cm horizontal offset, isocenter to surface distance (ISD) of 230 cm, and at 30 cm below the central axis, was made and showed the relative dose fall off measurement compared to the extrapolated data agreed within ± 1% of each other (see Figure 2a).

The horizontal uniformity measurements were combined with the output measured at different ISDs to get an estimate of the relative Rx dose that the patient would receive (Figure 2b), since the patient could not lie straight in both the AP/PA directions. There was an expected ± 25% difference in the prescription as the horizontal offset is ± 50 cm and depending on the ISD being 190 or 240 cm. The plan was to maintain the patient's treatment area within this offset and the ISD. The physician was informed of these estimated doses and approved. The physician was informed of these estimated doses and then proceeded with treatment after approval.

**FIGURE 3:**
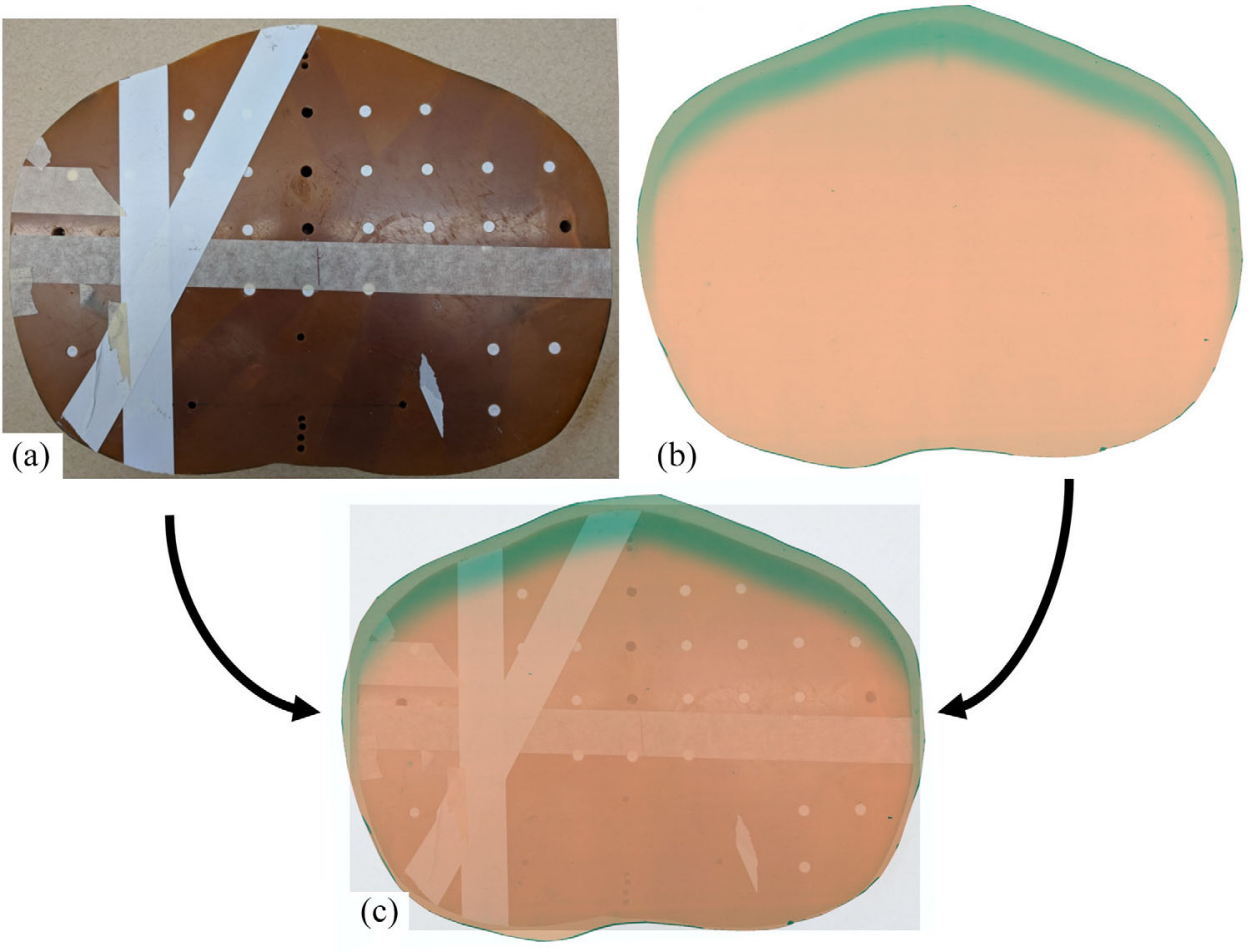
Top left (a) is a cross‐sectional view of the abdominal‐rando phantom used for PDD measurements with EBT3 Gafchromic film. On the top right, (b) shows an AP dual field irradiated film. On the bottom, shows (c) a fusion of the film and rando phantom to visually show the dose distribution of the dual fields.

To characterize the dose penetration and dose uniformity, EBT3 Gafchromic film was placed in an abdominal section of a rando anthropomorphic phantom (see Figure 3). The phantom with the film was placed at an ISD of 220 cm and was irradiated with an AP‐only dual field. The film was then analyzed in RIT software to measure the PDD at 0°, 30°, 60°, and 90° around the film. All the PDD curves were normalized to the 0° max dose to better understand the dose uniformity as well, because this is where the maximum dose was for the dual fields. The d_max_ for 0°, 30°, 60°, and 90° were 0.84, 0.62, 0.6, and 0.4 cm, respectively. The PDD at d_max_ for 30°, 60°, and 90° (normalized to PDD at d_max_) was 86.4%, 77.6%, and 32%, respectively.

### 2.2 Verification simulation and planning

During simulation, the patient surface was positioned in a single plane as best as possible while ensuring patient comfort and the ability to hold the position. Various ISDs were then measured for both the AP and PA fields. For the AP field, the ISD ranged from 192 to 210 cm, while for the PA field, the ISD ranged from 192 to 240 cm. This variation of PA and AP is due to the patient's flexation deformity secondary to his paraplegia. The average ISD was then used to calculate the monitor unit (MU), where

$$\text{MU}=\frac{\mathrm{Rx}\;\mathrm{dose}\;\left(\mathrm{cGy}\right)}{\mathrm{Output}\;\text{at average ISD}\;\left(\frac{\mathrm{cGy}}{\mathrm{MU}}\right)}$$

The average ISD measured for AP and PA fields was 215 and 226 cm, respectively.

### 2.3 In vivo dosimetry

In order to verify the prescription of 12 Gy in eight fractions, in vivo dose measurements were performed using MOSFET (Best Medical, Canada) detectors with no bolus placed over them. The MOSFET detectors were placed on the patient during the first full fraction (total of eight fields across two days). When the patient lay on his right side, the detectors were placed on the patient's anterior left chest, forearm, hip, upper thigh, and shin for the AP field, and then posterior left scapula, forearm, buttock, upper thigh, and lower leg for the PA field. While lying on the left side, MOSFETs were placed on the anterior right chest, forearm, hip, upper thigh, and shin for the AP field, and then the posterior right scapula, forearm, buttock, upper thigh, and lower leg for the PA field.

Table 1 shows the results of the measurements. All in‐vivo measurements were within ± 25% of the prescription dose, except those measured on the left buttock for the PA field with right side down, which was 30% above the Rx dose. This is most likely due to the patient's ability to hold the required position during treatment. All measurements are at the patient's surface.

**TABLE 1:** In vivo measurement for field sets 1 and 2. Prescription dose of 150 cGy per side.

	AP‐Patient Lying with Right Side Down			PA‐Patient Lying with Right Side Down		
		Measured Dose (cGy)	% Diff From Rx Dose		Measured Dose (cGy)	% Diff From Rx Dose
Field Set 1	Lt Chest	156	4%	Lt Scapula	166	10%
	Lt Forearm	128	−15%	Lt Forearm	178	18%
	Lt Hip	157	5%	Lt Buttock	195	30%
	Lt Upper Thigh	173	15%	Lt Upper Thigh	160	6%
	Lt Shin	145	−3%	Lt Lower Leg	144	−4%

	AP‐Patient Lying with Left Side Down			PA‐Patient Lying with Left Side Down		
		Measured Dose (cGy)	% Diff From Rx Dose		Measured Dose (cGy)	% Diff From Rx Dose
Field Set 2	Rt Chest	148	−2%	Rt Scapula	155	3%
	Rt Forearm	134	−11%	Rt Forearm	126	−16%
	Lt Hip	156	4%	Rt Buttock	170	14%
	Lt Upper Thigh	158	5%	Rt Upper Thigh	135	−10%
	Lt Shin	144	−4%	Rt Lower Leg	132	−12%

## 3 DISCUSSION & CONCLUSION

The patient was lying down on his left side down and right side down alternatively to ensure dose coverage of the opposite arm. Treatment time per fraction was about 40 min, including patient setup. He had a good response to TSET. Five months after treatment, he had reduced skin involvement with MF (4.2% of his skin surface, patch only disease).

With the dual‐beam setup and the patient lying down on his side, doses within 30% of the prescription dose were achievable. During each patient setup, it was ensured that the patient treatment area was within the planned ISD and distance from the central axis both vertically and horizontally. Wu et al.^[^ ^4^ ^]^ were able to achieve approximately 16% dose uniformity with the patient lying down supine and prone on the floor. In this technique, six dual fields were used. Kron et al.^[^ ^5^ ^]^ also described a lying down technique for pediatric patients up to 100 cm height, where dual six fields were used with the patient lying on the couch. The in vivo dosimetry showed a dose variation exceeding 20% of the prescription dose.

In conclusion, the decubitus TSET 4 dual field treatment for a paraplegic showed good response, and in vivo dose measurements were shown to be within ± 30% of the prescribed dose.

## AUTHOR CONTRIBUTIONS

All authors have made substantial contributions to the conception, design, and execution of the study.

- **Thomas Martin**: Performed the data collection, designed the experiments, and contributed to the interpretation of the data. Led the drafting of the manuscript.
- **Geoff Nelson**: Performed the data collection, designed the experiments, and contributed to the writing and revision of the manuscript.
- **David Gaffney**: Physician over patient treatment, guided clinical assessments, assisted in interpreting the results, and provided critical revisions to the manuscript.
- **Martin Szegedi**: Provided technical support for experimental procedures and patient treatments and contributed to the writing of the manuscript.
- **Christian Dial**: Provided technical support for experimental procedures and patient treatments and contributed to the writing of the manuscript.
- **Prema Rassiah**: Supervised the overall study, guided the analysis, and helped in revising the manuscript for intellectual content.

## CONFLICT OF INTEREST STATEMENT

All authors confirm that their financial interests, if any, have not influenced the content or interpretation of the research presented in this manuscript.

## Supporting information




Supporting Information
