# Supplementary material for: Modified total skin electron treatment for a paraplegic patient
Source: J Appl Clin Med Phys. 2025 Jul 14;26(7):e70162. doi: 10.1002/acm2.70162 (PMC12257338; doi:10.1002/acm2.70162)
Supplement: Supplementary file 1 — Supporting Information [file ACM2-26-e70162-s001.pdf]

Modified Total Skin Electron Treatment for a Paraplegic Patient

Thomas Martin, Ph.D.
Department of Radiation Oncology
University of Utah Huntsman Cancer Hospital
1950 Circle of Hope Dr.,
Salt Lake City, UT 84112
Thomas.martin@hci.utah.edu

Geoff Nelson
Department of Radiation Oncology
University of Utah Huntsman Cancer Hospital
1950 Circle of Hope Dr.,
Salt Lake City, UT 84112
Geoff.nelson@hci.utah.edu

David Gaffney
Department of Radiation Oncology
University of Utah Huntsman Cancer Hospital
1950 Circle of Hope Dr.,
Salt Lake City, UT 84112
David.Gaffney@hci.utah.edu

Christian Dial
Department of Radiation Oncology
University of Utah Huntsman Cancer Hospital
1950 Circle of Hope Dr.,
Salt Lake City, UT 84112
Christian.Dial@hci.utah.edu

Martin Szegedi
Department of Radiation Oncology
University of Utah Huntsman Cancer Hospital
1950 Circle of Hope Dr.,
Salt Lake City, UT 84112
Martin.Szegedi@hci.utah.edu

Prema Rassiah
Department of Radiation Oncology
University of Utah Huntsman Cancer Hospital
1950 Circle of Hope Dr.,
Salt Lake City, UT 84112
Prema.Rassiah@hci.utah.edu

Author Contribution Statement

All authors have made substantial contributions to the conception, design, and execution of the study.

- **Thomas Martin:** Performed the data collection, designed the experiments, and contributed to the interpretation of the data. Led the drafting of the manuscript.
- **Geoff Nelson:** Performed the data collection, designed the experiments, and contributed to the writing and revision of the manuscript.
- **David Gaffney**: Physician over patient treatment, guided clinical assessments, assisted in interpreting the results, and provided critical revisions to the manuscript.
- **Martin Szegedi** Provided technical support for experimental procedures and patient treatments and contributed to the writing of the manuscript.
- **Christian Dial**: Provided technical support for experimental procedures and patient treatments and contributed to the writing of the manuscript.
- **Prema Rassiah**: Supervised the overall study, guided the analysis, and helped in revising the manuscript for intellectual content.

Conflict of Interest Statement

All authors confirm that their financial interests, if any, have not influenced the content or interpretation of the research presented in this manuscript.

Acknowledgement Statement

We would like to acknowledge Huntsman Cancer Hospital for providing the necessary resources and equipment to conduct this case report study.
